# Pain Management and Cognitive Function Among Older Adults in China

**DOI:** 10.1093/geroni/igaa057.1698

**Published:** 2020-12-16

**Authors:** Xi Pan, Hongdao Meng

**Affiliations:** 1 Texas State University, San Marcos, Texas, United States; 2 University of South Florida, Tampa, Florida, United States

## Abstract

Chronic pain and cognitive decline are common age-related conditions affecting a large segment of older populations. Little is known about the pathway of cognitive functioning during the course of pain management in older adults. The current study aimed to examine the association between chronic body pain management and cognitive function over time among Chinese older adults. A total of 792 respondents aged 60 and above from urban and rural households in 28 provinces, 150 counties/districts, and 450 communities were selected from the China Health and Retirement Longitudinal Study (2013–2015). Cognitive function was measured in three domains: episodic memory, mental status, and global cognitive function. Difference-indifferences approach and mixed-effects linear regression models were employed to assess the association between chronic body pain management and cognitive function over time. Scores of mental status were found to decline slower by 0.49 unit (SE = 0.22, p < 0.05) in respondents who received pain management using analgesics, complementary and alternative medicine, or both from 2013 to 2015 after controlling for basic demographic and health confounders. Chronic pain management was associated with slower decline in domain-specific cognitive function, mental status over time. Findings of the study may contribute to understanding the mechanism of change in diverse cognitive abilities attributable to pain symptoms. More research is needed to elucidate the mediating effect of pain on cognitive decline, which could lead to testing of the impact of pain management on cognitive function among older population in both clinical and community settings.

